# Transition of microbiota in chicken cecal droppings from commercial broiler farms

**DOI:** 10.1186/s12917-020-02688-7

**Published:** 2021-01-06

**Authors:** Nachiko Takeshita, Takayasu Watanabe, Kasumi Ishida-Kuroki, Tsutomu Sekizaki

**Affiliations:** 1 Research Center for Food Safety, Graduate School of Agricultural and Life Sciences, The University of Tokyo, Yayoi 1-1-1, Bunkyo-ku, Tokyo, 113-8657 Japan; 2 Present Address: Department of Chemistry, Nihon University School of Dentistry, Kanda-Surugadai 1-8-13, Chiyoda-ku, Tokyo, 101-8310 Japan

**Keywords:** 16S rRNA amplicon sequence, Broiler chickens, Cecal dropping, *Campylobacter*, *Salmonella*

## Abstract

**Background:**

Chickens are major sources of human nutrition worldwide, but the chicken intestinal microbiota can be a source of bacterial infection. The microbiota has potential to regulate the colonization of pathogens by competitive exclusion, production of antimicrobial compounds, and stimulation of the mucosal immune system. But information on the microbiota in commercial broiler chickens is limited because of the difficulty of conducting studies at commercial farms. To obtain fundamental information that can be used to control pathogens in chickens, we determined the 6-week dynamics of microbiota in chicken cecal droppings from commercial broiler farms.

**Results:**

Cecal droppings from four chickens were collected once a week from 1 to 6 weeks of age at three commercial broiler farms. A total of 168 samples were collected from 7 flocks and subjected to 16S rRNA amplicon sequencing. Despite the farms have distinctly different climate conditions, the microbiota in the same growth stages were similar among farms. Moreover, as the chickens grew and the feed types were switched, the richness and diversity of the microbiota gradually increased and convergence of the composition of the microbiota was apparent. Notably, minor bacterial taxa (i.e. OTUs with relative abundance < 0.05%) within the microbiota were changed by the chicken age, switching of feed types, and presence of *Campylobacter*. In particular, the effects of switching of feed types on the microbiota were larger than the effects of age and *Campylobacter*.

**Conclusions:**

Irrespective of the locations of the farms, the microbiota of chicken cecum, especially minor bacteria, was successively changed more affected by feed types than by ages. Switching of feed types inducing the alteration of the microbiota may be associated with the colonization of pathogens in the chicken gut. These results will also help with extrapolation of studies in experimental animals to those in the commercial farms.

**Supplementary Information:**

The online version contains supplementary material available at 10.1186/s12917-020-02688-7.

## Background

Chickens are common domesticated animal worldwide, and chicken has become the leading meat consumed due to the short lifecycle and high feed conversion ratio [1]. On the other hand, chickens can also be sources of foodborne bacterial pathogens which can disseminate to humans or act as a pool for antimicrobial resistance and transmission [2–4]. Since the colonization of major foodborne pathogens, such as *Campylobacter* and *Salmonella enterica* serovars Enteritidis and Typhimurium, is often asymptomatic in broilers [5, 6], chickens with foodborne pathogens cannot be distinguished from the others. Therefore, it is necessary to prevent the colonization of foodborne pathogens in the gut to raise the pathogen-free chickens and supply safe chicken.

In recent years, an understanding of the mechanisms through which gut microbiota influence colonization of pathogens has become important for control of diseases for chickens. The chicken gut microbiota plays a key role in preventing invasion of pathogens by competitive exclusion, production of antimicrobial compounds, and stimulation of the mucosal immune system [7, 8]. In the gastrointestinal tract of chickens, the number and variety of bacteria are highest in the cecum, which contains up to 10^11^ bacteria/g [9, 10], and several studies have examined protection of chickens from pathogens by modification of the cecal microbiota [11–13]. However, the colonization of foodborne pathogens is ongoing problems in commercial farms. A major limitation is that most studies have used experimentally reared chickens, rather than commercial chickens, and the experimental models cannot fully mimic the actual conditions of commercial farms. In contrast, it is difficult to control the experimental conditions at farms, as complex environmental factors, such as biosecurity level, house type, and climate, may affect the composition of the chicken intestinal microbiota [14]. Thus, there have been few studies on the intestinal microbiota at commercial farms, with only two reports from one farm [15, 16], and the common factors affecting the cecal microbiota, excluding age, remain unclear.

In this study, we aimed to reveal the factor affecting cecal microbiota of commercial chickens and examined the microbiota of cecal droppings collected at 3 commercial broiler farms focusing on the differences between farms, ages, and feed types. Our findings will contribute to understand the fundamental and comprehensive feature of the cecal microbiota of commercial chickens and design experiments that mimic the conditions at commercial farms.

## Results

### Richness and diversity of the microbiota

**Fig. 1:**
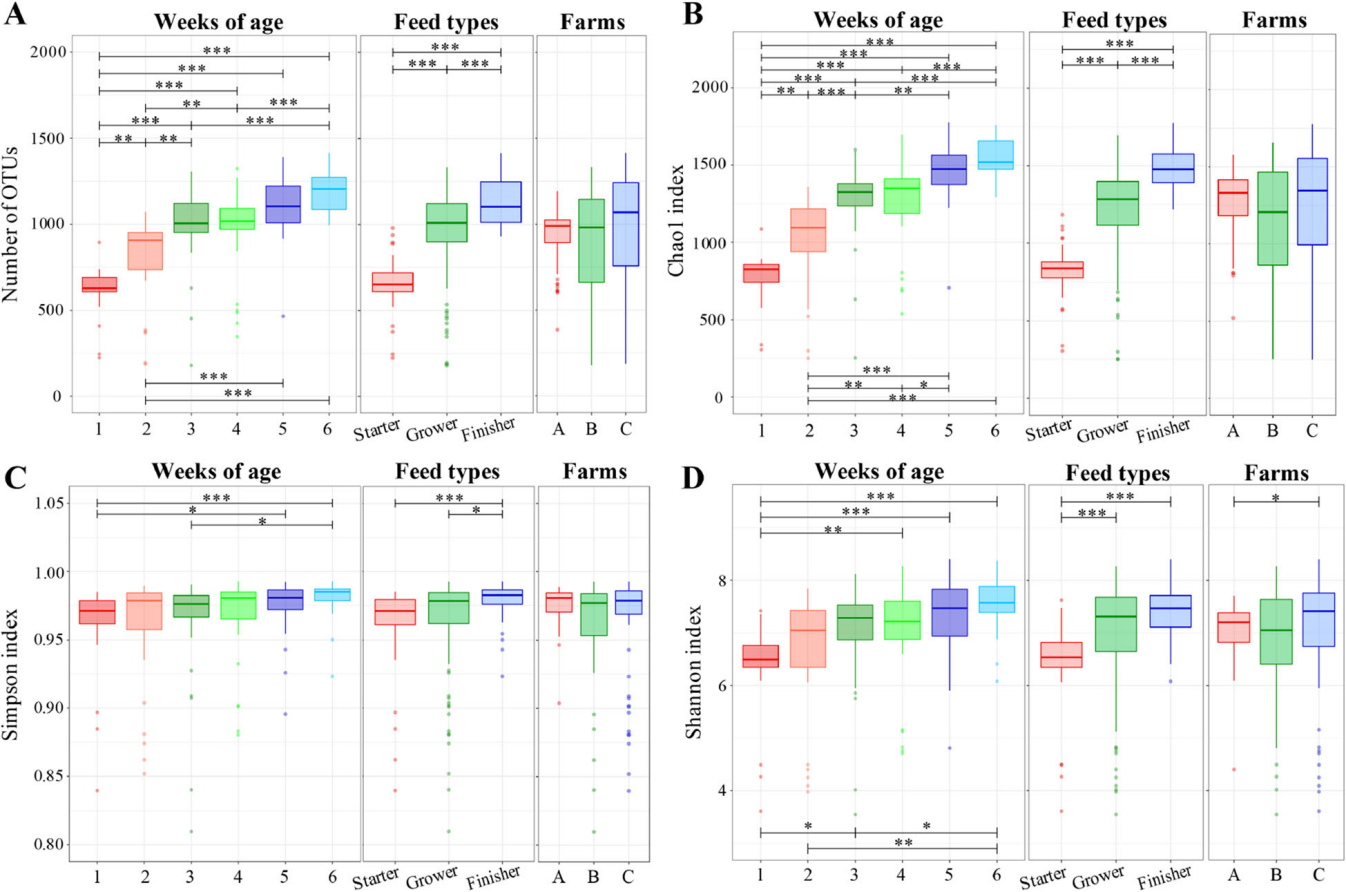
Differences in microbial richness and diversity among ages, feed types, and farms**.** Box plots show the number of OTUs (**a**), Chao1 index (**b**), Simpson index (**c**), and Shannon index (**d**). *P < 0.05; **P < 0.01; ***P < 0.001

On the basis of 97% sequence identity, a total of 3451 operational taxonomic units (OTUs) were obtained from 168 cecal dropping samples. The OTUs were assigned to 240 bacterial taxa; however, 26.7% (64 taxa) of the OTUs could not be assigned at the genus level. Rarefaction curves reached the saturation phase, and Good’s coverage was > 99% for all samples, which suggested that the reads obtained from the samples represented a sufficient number of sequences for analysis of the microbiota. The number of OTUs in cecal dropping samples significantly increased with the chicken age (Fig. 1a), and was also increased by switching of feed types (Fig. 1a). The Chao1 index differed significantly between all combinations of ages and feed types, except between samples collected in weeks 3 and 4, and weeks 5 and 6 (Fig. 1b). In contrast, the number of OTUs and Chao1 index showed no significant differences between farms (Fig. 1a and b). There were significant increases in the Simpson and Shannon indexes with the chicken age and with switching of feed types (Fig. 1c and d). The Shannon index also differed significantly between samples from farms A and C (Fig. 1d).

### Dominant bacterial taxa in the microbiota

**Fig. 2:**
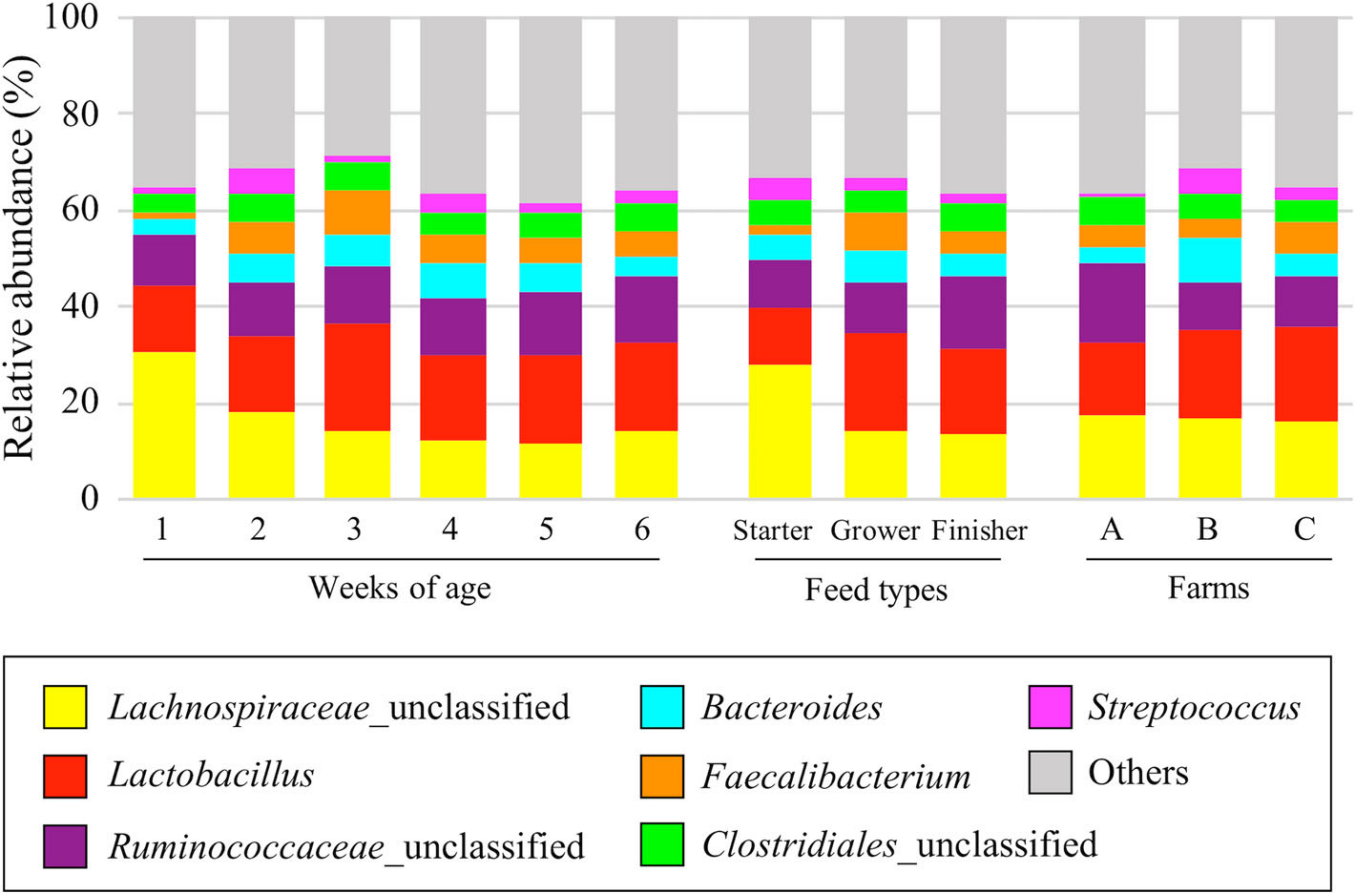
Genus-level distribution of the microbiota. Bacterial taxa with > 5% relative abundance in one or more of 12 categories (6 for age, 3 for feed type, and 3 for farms) are shown in different colors

The composition of the microbiota was examined for 6 age categories (1–6 weeks), 3 feed types (starter, grower, and finisher feeds), and 3 farms (farms A, B, and C), giving 12 categories in total. The major bacterial taxa and their proportions in cecal dropping samples were similar regardless of age, feed type, and farm, except that the level of *Lachnospiraceae*_unclassified significantly decreased in samples from weeks 1 to 2 and from use of starter to grower feeds (Steel-Dwass test, *P* < 0.001, Fig. 2). The bacterial taxa with > 5% average relative abundance in one or more of the 12 categories were *Lachnospiraceae_*unclassified (11.8 to 30.7%), *Lactobacillus* (11.8 to 21.9%), *Ruminococcaceae_*unclassified (9.7 to 16.3%), *Bacteroides* (3.1 to 9.5%)*, Faecalibacterium* (1.3 to 9.5%), *Clostridiales*_unclassified (4.2 to 6.0%), and *Streptococcus* (0.9 to 5.7%) (Fig. 2). *Lachnospiraceae*_unclassified, *Lactobacillus,* and *Ruminococcaceae_*unclassified were dominant, and these 3 bacterial taxa accounted for almost 40% of the microbiota (Fig. 2).

### Successive changes of the microbiota

**Fig. 3:**
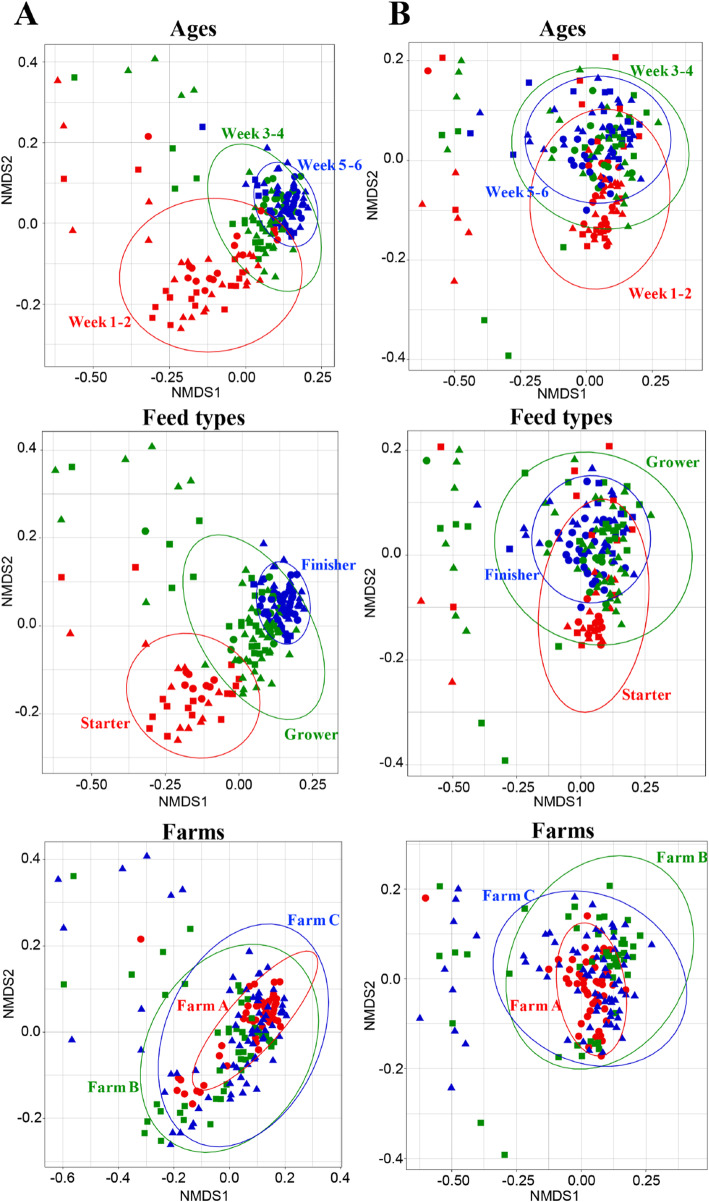
NMDS plots of the microbiota. NMDS of the microbiota based on unweighted (**a**) and weighted (**b**) UniFrac distance matrices of OTUs. Circles, squares, and triangles show each sample at farms **a**, **b** and **c**, respectively. The samples are indicated by different colors: red (weeks 1–2, starter feed, and farm **a**), green (weeks 3–4, grower feed, and farm **b**), and blue (weeks 5–6, finisher feed, and farm **c**). A 95% confidence ellipse is shown for each subgroup

Samples were divided into ages (weeks 1–2, 3–4, and 5–6), feed types (starter, grower, and finisher feed), and farms (farms A, B, and C), and non-metric multidimensional scaling (NMDS) was performed for these subgroups (Fig. 3). Using unweighted UniFrac distances, samples at weeks 5–6 were clustered more closely than those at weeks 1–2 and 3–4, resulting in a smaller 95% confidence ellipse for the samples at weeks 5–6 (Fig. 3a). Similarly, samples collected during use of finisher feed were clustered more closely than those collected during use of starter and grower feeds (Fig. 3a). Ellipses for samples at weeks 5–6 and with use of finisher feed almost overlapped with those for samples at weeks 3–4 and with use of grower feed, respectively. In contrast, ellipses for samples collected during use of finisher and starter feeds were separated from each other, despite the overlap of the ellipses for samples from weeks 1–2 and 5–6 (Fig. 3a).

Using weighted UniFrac distances, ellipses for samples at weeks 5–6 and with use of finisher feed were inside those for samples at weeks 3–4 and with use of grower feed, respectively (Fig. 3b). Ellipses for samples at weeks 1–2 and with use of starter feed partially overlapped with those for the other 2 categories (Fig. 3b). Ellipses for samples from each farm overlapped with each other in NMDS plots using unweighted and weighted UniFrac distances (Fig. 3a and b).

The compositional variation of the microbiota was measured by beta-dispersion based on unweighted and weighted UniFrac distances. Using unweighted UniFrac distances, the beta-dispersion analysis showed significant differences between samples at weeks 5–6 and those at other ages (Table 1), and between samples with use of finisher feed and other feed types. The compositional variation of the microbiota also differed significantly between samples from farms A and C. In the permutational multivariate analysis of variance (PERMANOVA), the R^2^ values for the percentage of variation explained by age, feed types and farms were 17.6, 19.7, and 6.47%, respectively (Table 1). With weighted UniFrac distances, the beta dispersion had no significant difference between samples from different ages, but did differ significantly between samples collected during use of grower and finisher feeds, and between samples from farm A and the other farms (Table 1). The R^2^ values for age, feed types and farms were 8.2, 8.8, and 5.9%, respectively (Table 1).

**Table 1:** Statistics for pairwise beta dispersion and PERMANOVA

Age, feed type, or farm	Beta dispersion	
	Unweighted UniFrac distance	Weighted UniFrac distance
Weeks 1–2 – Weeks 3–4	n.s.	n.s.
Weeks 1–2 – Weeks 5–6	*P* = 1.1e-5***	n.s.
Weeks 3–4 – Weeks 5–6	*P* = 4.9e-3**	n.s.
Starter – Grower	n.s.	n.s.
Starter – Finisher	*P* = 1.4e-2*	n.s.
Grower – Finisher	*P* = 8.0e-6***	*P* = 1.1e-2*
Farm A – Farm B	n.s.	*P* = 8.2e-3**
Farm A – Farm C	*P* = 6.5e-3**	*P* = 3.2e-3**
Farm B – Farm C	n.s.	n.s.
Age, feed type, or farm	PERMANOVA	
	Unweighted UniFrac distance	Weighted UniFrac distance
Ages	R^2^ = 0.1756 (*P* = 1e-04***)	R^2^ = 0.0822 (*P* = 1e-04***)
Feed types	R^2^ = 0.1974 (*P* = 1e-04***)	R^2^ = 0.0882 (*P* = 1e-04***)
Farms	R^2^ = 0.0647 (*P* = 1e-04***)	R^2^ = 0.0591 (*P* = 1e-04***)

*P < 0.05; **P < 0.01; ***P < 0.001

### Abundance of bacteria among ages and feed types

**Fig. 4:**
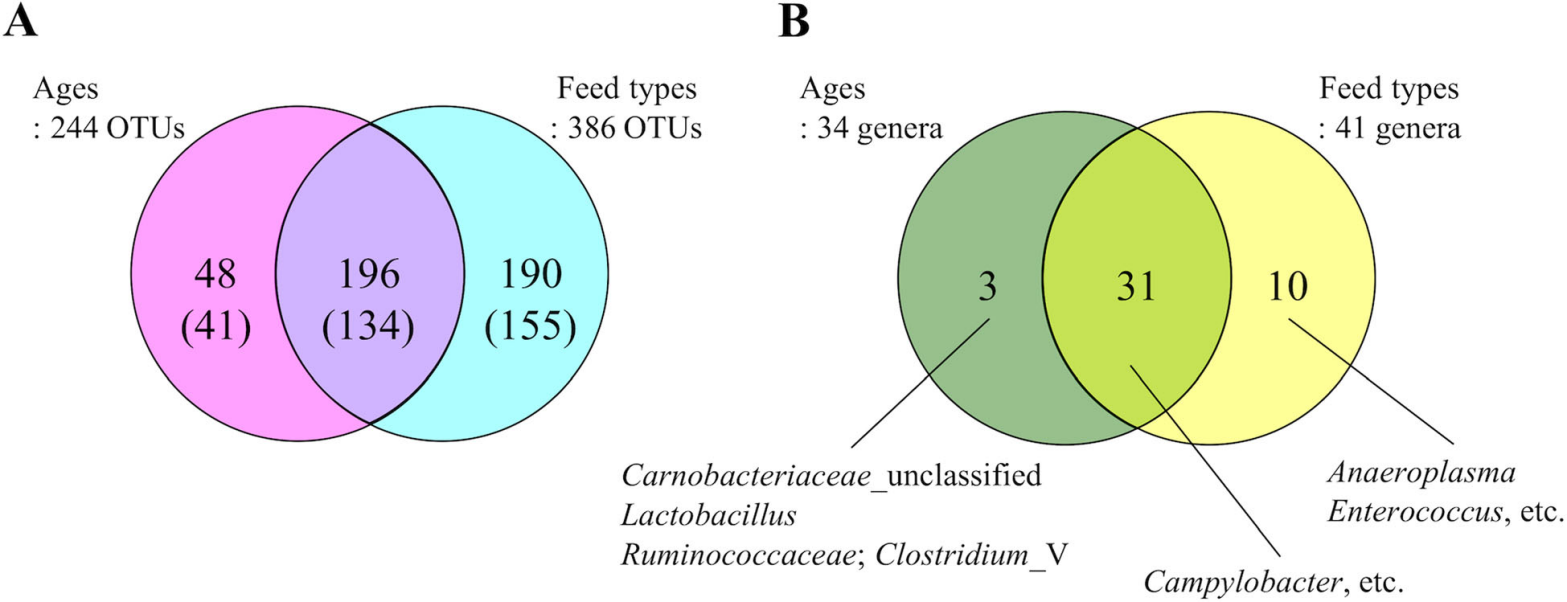
Venn diagrams representing the numbers of significantly different OTUs and bacterial genera. The numbers of significantly different OTUs (**a**) and bacterial genera (**b**) among ages or feed types (ANOVA, Bonferroni, *P* < 0.05). The numbers of minor OTUs (relative abundance in 168 samples < 0.05%) are shown in parentheses

OTUs with significantly different abundance among the subgroups of age and feed types are depicted in Fig. 4. Forty-eight and 190 OTUs were exclusively found in the age and feed type subgroups, respectively (Fig. 4a and Additional files 1 and 2: Tables S1 and S2), and the 48 OTUs involved 3 particular genera (Fig. 4b and Additional file 1: Table S1). In contrast, 10 genera, for example *Enterococcus*, had significantly different abundance among the subgroups of feed types, but did not vary among the subgroups of age (Fig. 4b and Additional file 2: Table S2). Among these genera, *Anaeroplasma* and *Enterococcus* (except for OTU 90) had decreased levels, and 6 genera increased with switching of feed types (Additional file 2: Table S2). There were 31 genera, including *Campylobacter*, that had significantly different abundances in both subgroups (Fig. 4b), but the relative abundance of the genus *Salmonella* did not differ in either subgroup. The relative abundance of these 31 genera were shown in Additional file 3: Table S3.

The number of minor OTUs (i.e. OTUs with relative abundance < 0.05%) was 3153 of 3451 OTUs (Additional file 4: Fig. S1), and the total average abundance of the minor OTUs accounted for 18.8% of that of the total OTUs. Among the OTUs with significantly different abundance among ages or feed types, 41 of 48 and 155 of 190, respectively, were minor OTUs (Fig. 4a and Additional files 1 and 2: Tables S1 and S2).

### Salmonella and campylobacter in the microbiota

The genus *Salmonella* was detected in 17 of 168 cecal dropping samples (Additional file 5: Table S4), which was too few to perform statistical analysis. The genus *Campylobacter* was detected in 21 of 168 samples, and 15 of them were detected at 6 weeks of age (Additional file 6: Table S5). There was no sample which was both positive for *Salmonella* and *Campylobacter.* In cecal dropping samples at 6 weeks of age, the relative abundance of 81 OTUs differed significantly by Welch *t*-test between *Campylobacter*-positive and -negative samples (Additional file 7: Table S6). Among the 81 OTUs, 64 (79.0%) were minor OTUs (relative abundance < 0.05%) (Additional file 7: Table S6).

## Discussion

In this study, we determined the 6-week dynamics of the composition of the microbiota in chicken cecal droppings. At commercial farms, chickens cannot be slaughtered for sampling because they are to be shipped as products. Therefore, cecal droppings were considered to be the best alternative to monitor the cecal microbiota because its diversity, richness and bacterial composition are reported to be similar to the microbiota of cecal contents [17]. To collect samples without disturbing the microbiota and to avoid stress on the chickens, we made the farmers collect cecal droppings. The composition of microbiota was similar with the previous study [17], and *Lachnospiraceae_*unclassified, *Lactobacillus*, and *Ruminococcaceae_*unclassified were dominant in the microbiota (Fig. 2), indicating that no obvious contamination was occurred at the sampling.

The richness and diversity of the microbiota increased with the chicken age (Fig. 1), as reported previously [18–21]. The richness and diversity were also increased by switching of feed types (Fig. 1), which reflects the effects of age because feed types were switched with growth of the chickens. In contrast, differences in microbial richness and Simpson’s diversity were not observed among farms (Fig. 1). Moreover, the R^2^ value of PERMANOVA for the farms was lower than those for ages and feed types (Table 1), indicating that the different farms had less effect on microbial variation. On the other hand, the differences of geographic location among the farms were explained by the significant difference in beta dispersion (Table 1). Farm A, which is located in the northern part of Japan, is about 1300 km from farms B and C, which are located in the southern part of Japan. Northern and southern Japan have distinct climates with different ranges of temperature, different amounts of total rain and snow fall, and different levels of humidity. The broiler house types also differed between farm A (windowless type for preparing for cold weather) and the other farms (open-sided type for preparing for hot weather). Previous studies have suggested that chickens of the same age have differences in their composition of cecal microbiota due to geographic location [22, 23], climate [18], and season [24], whereas significant differences caused by feed additives were not found [25]. Our findings indicate only small effects of location and climate of farms on the microbiota, with these effects being less important than those of age or feed types.

The composition of the microbiota converged with increased age in the NMDS plot based on unweighted UniFrac distances (Fig. 3a), which is consistent with previous studies [13, 26]. Moreover, the composition of the microbiota converged with switching of feed types (Fig. 3a). These observations suggest that the microbiota may become stable and show less variation between individual chickens as a consequence of acquisition and replacement of new bacteria through growth. In contrast, using weighted UniFrac distances, a difference in composition of the microbiota was not clear in all samples (Fig. 3b). In these distances, low abundance taxa have a low impact on the total distance metrics [27]. Therefore, weighted UniFrac distances are mainly affected by OTUs comprising the major bacterial taxa whose abundance was similar regardless of age and feed type (Fig. 2). This finding suggests that minor bacterial taxa mainly shifted within the microbiota with increased age and switching of feed types. Consistent with this idea, the microbiota was mostly minor OTUs (Additional file 4: Fig. S1), and the abundance of these OTUs differed significantly among feed types as well as ages (Fig. 4a). Therefore, it is plausible that the composition of the microbiota changes due to acquisition and replacement of minor bacterial taxa throughout the growth of chickens.

This study also showed that chicken age and switching of feed types may have contributed differently to the changes in the composition of the microbiota. Despite some differences in feeding schedules among the farms, switching of feed types was related to increased age. This relationship makes comparisons complex, and thus, the effects of switching of feed types on the microbiota have yet to be completely clear. NMDS plots based on unweighted UniFrac distances suggested that changes in this composition have coincided with switching of feed types, rather than age (Fig. 3a). Furthermore, a Venn diagram showed many significantly different OTUs among feed types (Fig. 4a). Thus, the effects of switching of feed types on the microbiota seemed to be larger than those of age.

The abundance of minor OTUs was changed by the presence of *Campylobacter*, as shown by a Welch *t*-test (Additional file 7: Table S6). There was a significantly lower abundance of *Lactobacillus* and a higher abundance of *Streptococcus* in *Campylobacter*-positive samples than in negative samples (Additional file 7: Table S6), as also reported previously [28, 29]. However, most minor OTUs were not characterized, and the correlation between the minor OTUs and colonization of *Campylobacter* was not clear. Previous evidence suggested that minor bacteria had a beneficial influence on host animals and breeding success [30, 31]; thus, these bacteria should not be ignored due to their influence on host health and their beneficial role in chickens.

The relative abundance of *Campylobacter* was significantly increased by age and by switching of feed types (Fig. 4b). Several studies have shown an association between age and colonization of *Campylobacter* [32–34]. However, little is known about the effects of switching of feed types, with only one report [20]. Interestingly, the amount of *Enterococcus* was decreased by switching of feed types (Additional file 2: Table S2). *Enterococcus faecium* and *Enterococcus faecalis* cause enterococcal infections in poultry and humans, and acquisition of resistance to antibiotics by these bacteria is a serious problem [35, 36]*.* In commercial farms, *Enterococcus cecorum* causes diseases of chickens that lead to economic losses for farmers [37]. Early establishment of a mature gastrointestinal microbiota has been associated with prevention of colonization by some pathogens [38, 39]. Thus, a stable microbiota with high diversity and low variation during use of finisher feed may have decreased the *Enterococcus* level (Figs. 1 and 3a). Taken together, these results suggest that feed types have a major effect on changes of abundance of specific bacteria, including pathogens, within 50 days of ages. Switching of feed types that induces the alteration of the microbiota which could affect colonization of pathogens in the chicken gut, although other various factors, such as immunity of the chickens, gut health, broiler strains, climatic conditions, and the surrounding environment could also affect the colonization. As the microbiota in a certain growth stage was similar among farms, our results are not limited to this study and may represent the general features of the microbiota in chicken cecal droppings.

## Conclusions

We obtained fundamental information on the microbiota in chicken cecal dropping from commercial farms. The composition of the microbiota seemed to change due to acquisition and replacement of minor bacterial taxa with increasing chicken age, switching of feed types, and presence of *Campylobacter*. The composition of minor bacteria may have affected colonization of pathogens in the gut, therefore, understanding the role of minor bacteria is considered important. Especially, switching of feed types may have a major impact on changes of the composition of minor bacteria in the microbiota. Therefore, replacement of feed types throughout the growth of chickens may be an important factor for mimicking commercial broiler chickens under experimental conditions. Furthermore, it may be better to start administration of probiotics during use of starter feed before the chicken cecal microbiota becomes diverse and stable. Further studies are needed to identify the effects of switching of feed types on the chicken cecal microbiota for utilizing the feeds to prevent colonization by pathogens.

## Methods

### Animals and sample collection

From October 2016 to January 2018, cecal dropping samples from 4 clinically healthy broiler chickens (Chunky breed) per flock were collected by farmers once a week from 1 to 6 weeks of age. Four cecal droppings in each sampling was the maximum number that the farmers could collect. The cecal droppings were collected randomly from 2 flocks at farms A and B, and from 3 flocks at farm C (Additional file 8: Fig. S2), and a total of 168 samples were obtained. Farm A is located in Tohoku, in the north of Japan’s main island, and farms B and C are located in Kyushu, the southern island of Japan. The area of chicken houses and the approximate number of chickens per flock were as follows: farm A, 463 m^2^ and 10,000 chickens; farm B, 660 m^2^ and 9000 chickens; farm C, 1040 m^2^ and 17,000 chickens. The type of broiler house was windowless in farm A, and open-sided in farms B and C. Each owner gave consent for collection of samples. At the 3 farms, all chickens were reared for 50 days until shipment, except that parts of 2 flocks at farm A were shipped to another client at 33 days of age (Additional file 8: Fig. S2). After all chickens were shipped, the houses were cleaned and disinfected, and the litter between the flocks within the same house was changed at the 3 farms.

Poultry feeds were categorized into 3 types: starter, grower, and finisher feeds. These were fed to chickens depending on growth, and the ages of switching of feeds varied among the farms (Additional file 8: Fig. S2). According to information from the farms, the amount of protein in chicken feeds decreased in the order of starter, grower, and finisher feeds, whereas the amount of lipids in the feeds increased in the same order. The detailed information on nutrition contained in the feeds was confidential.

The cecal droppings were collected by the same farmers at each farm, based on our instructions for sampling. For collection of cecal droppings, the farmers did not touch the animals and simply picked up the cecal droppings. The Institutional Animal Care and Use Committee of the Graduate School of Agriculture and Life Sciences, the University of Tokyo, confirmed that the study did not require approval. Each cecal dropping was immediately collected using a sterilized 150-mm polypropylene spatula (As One, Osaka, Japan) and immersed in 500 μl of RNA*later* Stabilization Solution (Thermo Fisher Scientific, Waltham, MA) in a conical tube to prevent bacterial growth and degradation of DNA. Cecal dropping samples were transported to our laboratory under refrigeration within 2 days and stored at − 20 °C until use.

### Extraction of DNA from cecal dropping samples

A total of 168 samples were used for DNA extraction. Frozen samples were thawed and centrifuged at 13,000×*g* for 5 min at 4 °C. The pellet in each tube was washed twice with sterile 0.85% saline [40] and total DNA was extracted using a PowerFecal® DNA Isolation kit (Qiagen, Hilden, Germany). To increase the extraction efficiency, 400 μl of 0.5-mm diameter zirconia beads (Toray, Tokyo, Japan) and two 5-mm diameter zirconia beads (Toray) were used instead of the beads in the kit [40], with a μT-12 bead crusher (Taitec, Saitama, Japan). Total DNA was eluted in 100 μl of the elution buffer in the kit and stored at − 20 °C until use. The DNA concentration was measured with a Quantus™ Fluorometer with a QuantiFluor® dsDNA System (Promega, Madison, WI).

### 16S rRNA gene amplicon sequencing

The V3-V4 regions of 16S rRNA genes in the extracted DNA were amplified with S-D-Bact-0341-b-S-17 (5′-TCG TCG GCA GCG TCA GAT GTG TAT AAG AGA CAG CCT ACG GGN GGC WGC AG-3′), and S-D-Bact-0785-a-A-21 (5′-GTC TCG TGG GCT CGG AGA TGT GTA TAA GAG ACA GGA CTA CHV GGG TAT CTA ATC C-3′) primers [41], include the Illumina overhang adapter sequence (Illumina, San Diego, CA). Prior to PCR amplification, the amount of total bacterial genome DNA contained in the extracted DNA was estimated by quantitative real-time PCR [40]. The 25 μl reaction volume in the PCR contained 12.5 μl of 2× KAPA HiFi HotStart ReadyMix DNA polymerase (Kapa Biosciences, Woburn, MA), 0.2 μM of forward and reverse primers, and 12.5 ng of DNA template (amount of total bacterial DNA). The PCR conditions were as follows: 95 °C for 3 min; 25 cycles at 95 °C for 30 s, 55 °C for 30 s, and 72 °C for 30 s; and a final extension step at 72 °C for 5 min. The PCR amplification and the quality of the PCR products were checked using an Agilent 2100 Bioanalyzer (Agilent Technologies Japan, Tokyo, Japan). The products were purified with Agencourt AMpure XP beads (Beckman Coulter Inc., Brea, CA). Dual indexes and sequencing adapters were attached by subsequent PCR using a Nextera XT Index Kit (Illumina). The indexed products were further purified using AMpure XP beads (Beckman Coulter Inc.) and checked with the Bioanalyzer (Agilent Technologies Japan). The prepared libraries were quantified by qPCR with a Library Quantification Kit for Illumina (Kapa Biosciences). Libraries of equimolar DNA molecules were pooled and diluted in hybridization buffer. Sequencing was performed using the 2 × 300 bp paired-end method on the MiSeq platform with a MiSeq v3 Reagent Kit (Illumina) and a 50% phiX spike.

### Sequence data processing and taxonomy assignment

Fastq reads were processed using the analysis pipeline Illinois Mayo Taxon Organization from RNA Dataset Operations ver. 2.0.3.2 [42] with default parameters, except for the following parameters in Trimmomatic: LEADING:20, TRAILING:20, and MINLEN:180. Trimmomatic was used to trim low-quality sequences. For ensuring use of high-quality reads when assigning OTU representation, singletons and chimeric reads were discarded in the pipeline. Mothur [43] was used for OTU clustering at 100% sequence identity, and the OTUs were sorted by cluster size and processed in USEARCH using the UPARSE algorithm to detect OTU representatives using a de novo OTU picking strategy. OTUs were picked and assigned using the Ribosomal Database Project (RDP) naive Bayesian classifier [44] at 97% similarity against the RDP database (v.11; Ribosomal Database Project, Michigan State University, East Lansing, MI) [45]. The number of sequences was normalized to 25,000 for each sample with the core_qiime_analyses.py script from Quantitative Insights Into Microbial Ecology (QIIME) ver. 1.9.1 [46] and used in the following analyses; this means that the detection limit was relative abundance of 0.004% in each sample. In addition, a sample containing at least one sequence classified as *Salmonella* or *Campylobacter* was considered positive for these bacteria.

### Statistical analysis

Indices of Chao1, Simpson, Shannon, and Good’s coverage of samples were calculated and rarefaction curves were constructed using QIIME. Significant differences (*P* < 0.05) of the alpha-diversity indices between samples were examined by a nonparametric multiple comparison test (Steel-Dwass) using the pSDCFlig function in the NSM3 package in R [47].

From the downstream analysis, the sequencing data from cecal dropping samples were divided into subgroups by age of chickens (weeks 1–2, 3–4 and 5–6), feed types (starter, grower, and finisher feed) or farms (farms A, B, and C). The unweighted and weighted UniFrac distance [48] for each subgroup was calculated from the normalized composition of microbiota in cecal dropping samples using QIIME. NMDS based on the obtained distance matrix was conducted using the vegan package in R, and visualized with 95% confidence ellipses. The ellipses were drawn using ggplot2 stat_ellipse (type = “norm”) function, and represent the 95% confidence interval of the multivariate *t*-distribution.

Beta-dispersions measuring the compositional variation of the microbiota among subgroups were analyzed using the vegan package in R. The average distance of each sample to the centroid of the subgroup in multivariate space was calculated based on unweighted and weighted UniFrac distances. A Tukey honestly significant difference test was then conducted for pairwise comparisons of mean dispersions among subgroups. Differences in bacterial communities among the subgroups were evaluated with PERMANOVA with 9999 random permutations using the vegan package.

To identify significant differences in OTU abundances among ages and feed types (Bonferroni *P* < 0.05), an analysis of variance (ANOVA) was conducted using QIIME, and Venn diagrams were generated to compare the OTUs between ages and feed types. OTUs with an average relative abundance in 168 samples < 0.05% were defined as minor OTUs, and the frequency distribution was created for the number of OTUs.

A Welch *t*-test was performed to test the significance of differences between samples with and without *Campylobacter* (*P* < 0.05).

## Supplementary Information

**Additional file 1.** OTUs which relative abundances were significantly different among the subgroups of age but not among the subgroups of feed type.

**Additional file 2.** OTUs which relative abundances were significantly different among the subgroups of feed type but not among the subgroups of age.

**Additional file 3.** Bacterial genera which relative abundances were significantly different both among the subgroups of age and feed type.

**Additional file 4: Fig. S1.** Frequency distribution of the relative abundance of OTUs. Minor OTUs (relative abundance in 168 samples< 0.05%) are shown in orange. Major OTUs (relative abundance in 168 samples≥0.05%) are shown in blue.

**Additional file 5 **Prevalence of *Salmonella* in broiler chickens.

**Additional file 6 **Prevalence of *Campylobacter* in broiler chickens.

**Additional file 7 **OTUs which relative abundances were significantly different between *Campylobacter*-positive and -negative samples.

**Additional file 8: Fig. S2.** Time points of sampling and feeding schedules. Triangles (▼) and squares (■) show time points of sampling and thinning, respectively. Feed types are indicated by different colors: red (starter feed), green (grower feed), and blue (finisher feed), and are separated by circles (●).

## Data Availability

Datasets generated by 16S rRNA gene amplicon sequencing in this study have been deposited in the DNA Data Bank of Japan under accession number DRA007956.

## Abbreviations

ANOVA: analysis of variance
NMDS: non-metric multidimensional scaling
OTU: operational taxonomic unit
PERMANOVA: permutational multivariate analysis of variance
QIIME: Quantitative Insights Into Microbial Ecology
RDP: Ribosomal Database Project
