# Supplementary figures and images for: Transition of microbiota in chicken cecal droppings from commercial broiler farms
Source: BMC Vet Res. 2021 Jan 6;17:10. doi: 10.1186/s12917-020-02688-7 (PMC7789685; doi:10.1186/s12917-020-02688-7)

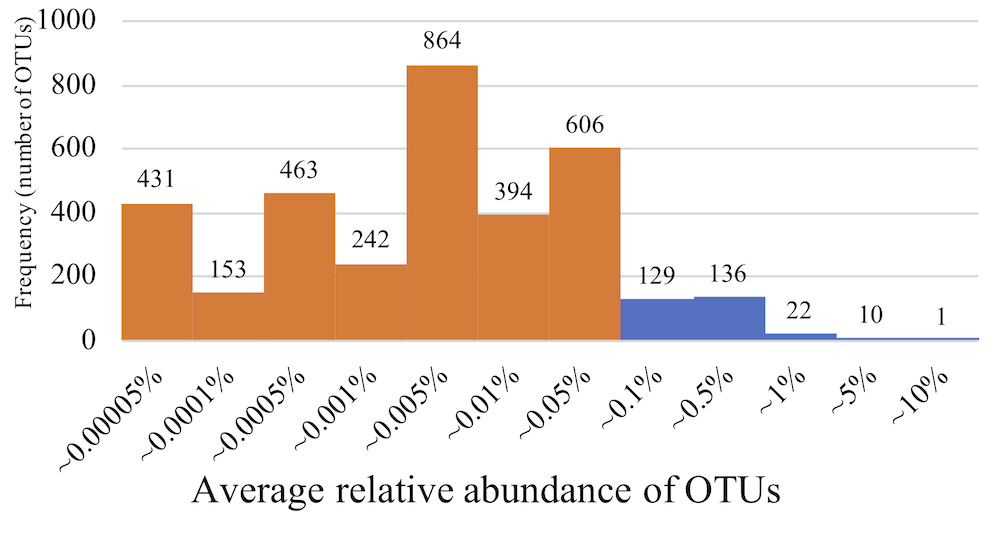

Supplement: Supplementary file 4 — Additional file 4: Fig. S1. Frequency distribution of the relative abundance of OTUs. Minor OTUs (relative abundance in 168 samples< 0.05%) are shown in orange. Major OTUs (relative abundance in 168 samples≥0.05%) are shown in blue. [file 12917_2020_2688_MOESM4_ESM.tiff]

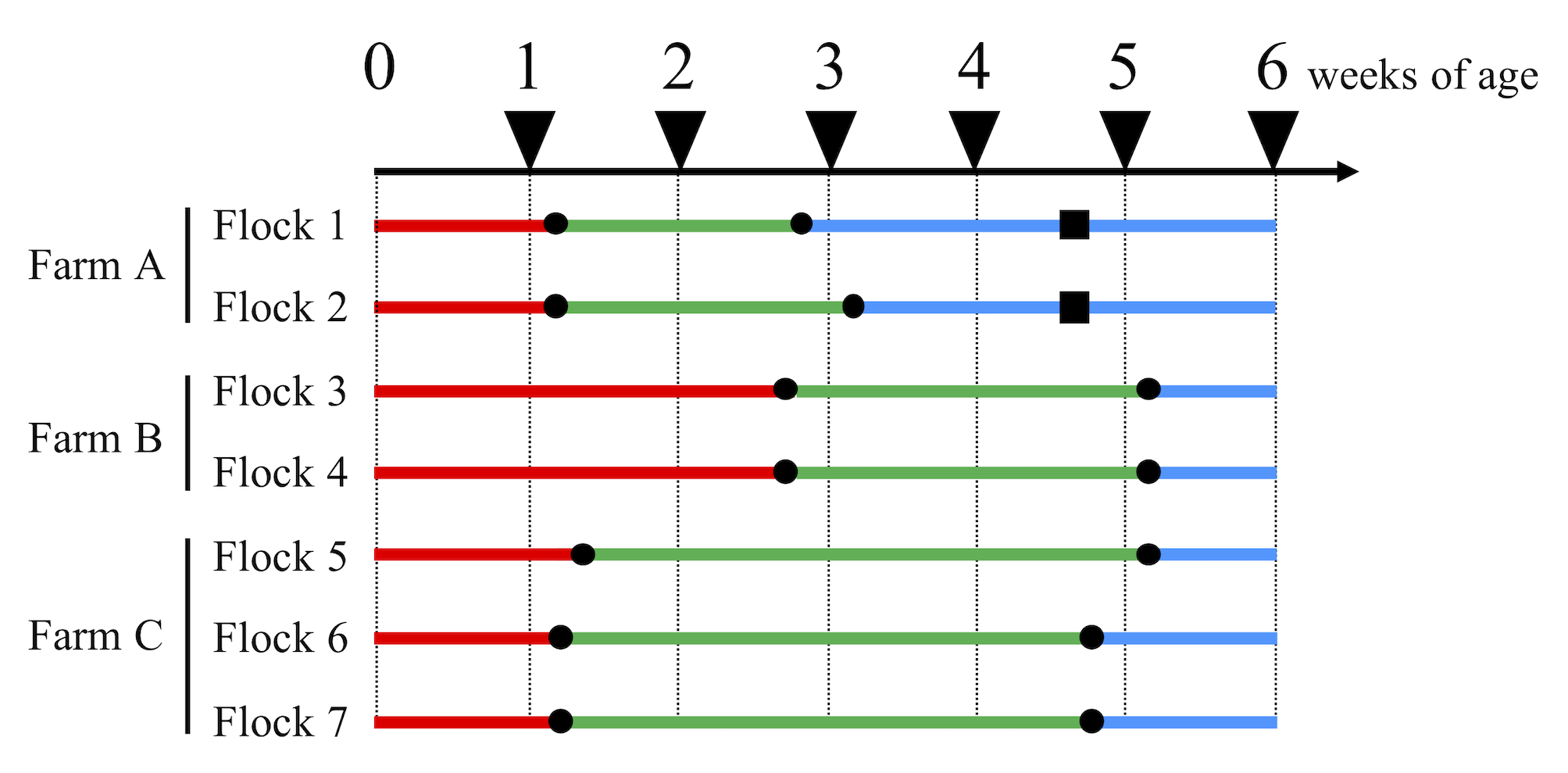

Supplement: Supplementary file 8 — Additional file 8: Fig. S2. Time points of sampling and feeding schedules. Triangles (▼) and squares (■) show time points of sampling and thinning, respectively. Feed types are indicated by different colors: red (starter feed), green (grower feed), and blue (finisher feed), and are separated by circles (●). [file 12917_2020_2688_MOESM8_ESM.tiff]
